# Disorder in a two-domain neuronal Ca^2+^-binding protein regulates domain stability and dynamics using ligand mimicry

**DOI:** 10.1007/s00018-020-03639-z

**Published:** 2020-09-16

**Authors:** Lasse Staby, Katherine R. Kemplen, Amelie Stein, Michael Ploug, Jane Clarke, Karen Skriver, Pétur O. Heidarsson, Birthe B. Kragelund

**Affiliations:** 1 Department of Biology, University of Copenhagen, Ole Maaloes Vej 5, 2200 Copenhagen, Denmark; 2 Rigshospitalet, Finsen Laboratory, Ole Maaloes Vej 5, 2200 Copenhagen, Denmark; 3 Biotech Research and Innovation Centre, University of Copenhagen, Ole Maaloes Vej 5, 2200 Copenhagen, Denmark; 4 Department of Chemistry, University of Cambridge, Cambridge, CB2 1EW UK; 5 Department of Biochemistry, Science Institute, University of Iceland, 107 Reykjavík, Iceland

**Keywords:** NCS-1, Frequenin, IDP, EF-hand, Protein folding, Order–disorder interplay

## Abstract

Understanding the interplay between sequence, structure and function of proteins has been complicated in recent years by the discovery of intrinsically disordered proteins (IDPs), which perform biological functions in the absence of a well-defined three-dimensional fold. Disordered protein sequences account for roughly 30% of the human proteome and in many proteins, disordered and ordered domains coexist. However, few studies have assessed how either feature affects the properties of the other. In this study, we examine the role of a disordered tail in the overall properties of the two-domain, calcium-sensing protein neuronal calcium sensor 1 (NCS-1). We show that loss of just six of the 190 residues at the flexible C-terminus is sufficient to severely affect stability, dynamics, and folding behavior of both ordered domains. We identify specific hydrophobic contacts mediated by the disordered tail that may be responsible for stabilizing the distal N-terminal domain. Moreover, sequence analyses indicate the presence of an LSL-motif in the tail that acts as a mimic of native ligands critical to the observed order–disorder communication. Removing the disordered tail leads to a shorter life-time of the ligand-bound complex likely originating from the observed destabilization. This close relationship between order and disorder may have important implications for how investigations into mixed systems are designed and opens up a novel avenue of drug targeting exploiting this type of behavior.

**Electronic supplementary material:**

The online version of this article (10.1007/s00018-020-03639-z) contains supplementary material, which is available to authorized users.

## Introduction

Many studies describe the relationship between protein structure and function, with the underlying premise that the ability of a protein to perform a specific biological function is encoded in its precise three-dimensional (3D) fold [1–3]. While this view has been widely accepted, the dynamic properties of proteins have also been shown to play as large and decisive a role as structure in determining and modulating function [4, 5]. Intrinsically disordered proteins (IDPs) are involved in a large number of central biological processes [6, 7], yet they do not attain a specific 3D structure but instead consist of a broad and fluctuating ensemble of near isoenergetic conformations. Still, in a large fraction of proteins, order and disorder co-exist [8], but it is currently not understood how the function of an ordered segment is affected by the proximity of, and physical link to, disordered regions and vice versa. As most human proteins contain disordered elements [9, 10] the question of how the characteristics and properties of folded domains change when they exist in the context of a disordered chain, remains important and has broad implications [11, 12].

Adjoining disorder in a peptide chain may affect the ability of a globular domain to fold correctly or to bind specific partners, or it may affect the conformational ensemble of the disordered chain [13]. These effects could be rooted in forming non-native interactions between the ordered and disordered chains, hindered diffusion of the chain along a coordinate, a restricted folding landscape or electrostatic attraction between the two parts. The effects may be either positive or negative, as non-native interactions may increase the likelihood of misfolding and/or chain accessibility, whereas restrictions along the folding landscape may preclude the formation of stable aggregates. Indeed, experiments on fusion proteins have shown that addition of a disordered tail to a well-folded domain affects overall structural properties [14]. It is likely that proteins in which order and disorder have co-evolved have an intricate interplay of their ordered and disordered elements, as recently suggested from entropic effects of disorder tuning enzymatic function [15]. In this work, we set out to assess the interplay of order and disorder in a neuronal calcium sensor protein.

**Fig. 1:**
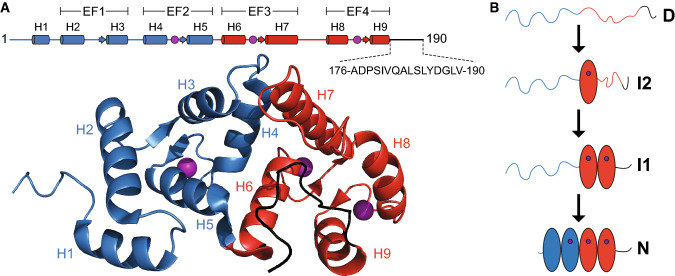
**a** Structure of NCS-1 (PDB 2LCP) showing the N-domain (blue), C-domain (red), disordered tail (black) and Ca^2+^ atoms (magenta) [33]. The sequence of the disordered C-terminal tail and the calcium binding loops are indicated. **b** Folding trajectory of NCS-1 as previously determined by single-molecule force experiments [37]

The EF-hand family of calcium binding proteins shares a base structure of four EF-hands in two paired domains [16, 17], and several NMR and crystal structures are available. Multiple homologues exist across species as well as within a single organism [18, 19], but these paralogs have little functional redundancy and their sequences vary, particularly in the termini [20]. Members of this family carry out diverse functions such as ion buffering in the cytosol [20], signal transduction [21], muscle contraction and neurotransmission [22–24], and many are implicated in different disease states [25–27]. Neuronal calcium sensor-1 (NCS-1; also called frequenin) is a 190-residue protein (Fig. 1a), belonging to the NCS subfamily of EF-hands that potentiates neurotransmitter release [24, 28, 29] and it has been linked to several human disorders including autism and schizophrenia [26, 30]. Its N-terminus is myristoylated in vivo, facilitating interactions with membrane-associated partners via insertion of the myristoyl group into the lipid bilayer [31, 32]. There is some debate as to whether this insertion is permanent or whether NCS-1 cycles between the membrane and the cytosol [31]. NCS-1 has two EF-domains, each consisting of a pair of EF-hands, which are joined by a short four-residue linker (R94-G95-T96-L97) sometimes referred to as the hinge region [33, 34] and with allosteric interdomain interactions [35]. In contrast to e.g. calmodulin, the two domains of NCS-1 form intimate contacts, making the folding behavior highly complex as observed by single molecule force spectroscopy [36, 37]. Folding occurs via two intermediates with the EF-hands of the C-domain forming individually, followed by concurrent formation of the two N-terminal EF-hands of which only EF2 is calcium binding competent (Fig. 1b). This complexity has so far precluded investigation of the system by bulk kinetic experiments as calcium-dependent misfolding also occurs [37].

A C-terminal tail of varying length is common to the subfamily of NCS proteins. These tails assume different conformations in the 3D structures, depending on whether crystalized in co-additive media or solved in solutions [33, 38–40] (Table S1). Generally, a hydrophobic binding cleft opposite to the calcium binding loops is formed across the N- and C-domains, which in some cases, as in recoverin, may be occupied by the myristoyl group [40], whereas for others it is filled with co-additives such as PEG when crystallized [41] or, as in the case of NCS-1, its dynamic C-terminal tail [33]. The cleft acts as a binding site for a large number of partners, and the structure of NCS-1 in complex with one such partner, phosphatidylinositol-4-kinase (PIK1), shows that the disordered C-terminal tail is displaced from the cleft [42].

In solution, the C-terminal 15-residue tail of NCS-1 is dynamic and adopts a multitude of conformations, serving as a moving lid for the cleft in the absence of ligands. Loss of this tail has a dramatic effect on the dynamics of NCS-1, an effect which is also observed with an autism-related mutation in NCS-1 (R102Q) [30, 33]. Tail-dependent effects have been identified in other calcium-binding proteins. In a calmodulin variant from rice, the disordered C-terminal extension affected both the stability of the apo-state as well as the order of Ca^2+^-ion binding, and the tail was speculated to increase target selectivity [43]. Similarly, tail-dependent binding specificity was seen for calcium and integrin-binding protein 1 (CIB1) where the C-terminal disordered tail interacted weakly with the ligand-binding hydrophobic channel and was displaced upon ligand binding [44]. Thus, calcium-binding proteins appear to be associated with disorder, and disordered tails can have various effects on their structure, function and stability.

Here we investigate the connection between order and disorder in NCS-1 by scrutinizing the properties of the disordered tail and its effects on the rest of the protein and its ligand binding capabilities. We have measured the impact of a set of tail truncations, amino acid substitutions, and tail-swaps on stability and structure of the globular part of NCS-1, and used sequence analysis across species to identify sequence elements responsible for long-range communication. We find that the tail strongly influences the stability of the distal N-domain, and although it appears that diverse tails can fulfil the task, there exists a degenerate sequence motif linking the tails to the N-domain. This same motif appears in NCS-1 binding partners incorporated in a helix scaffold, hence providing the tail with ligand-mimicking properties. In the absence of the tail, the affinity for a fragment of the binding partner PIK1 is decreased substantially, driven primarily by shortening the half-life of the complex, likely through the destabilization effect.

## Results

### A C-terminal tail is vital for the stability of N-domain

**Fig. 2:**
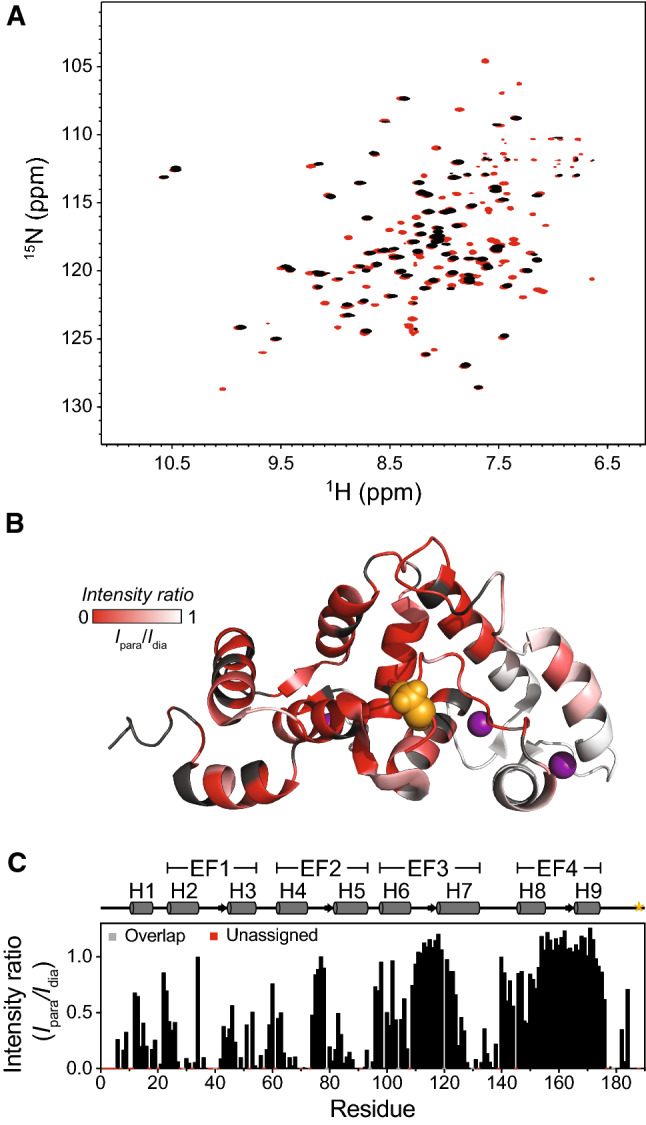
Paramagnetic relaxation enhancement effect in NCS-1_C38S G188C_. **a** Comparison of BEST-TROSY spectra of MTSL labeled NCS-1_C38S G188C_ before (black) and after (red) reduction with 5 mM ascorbic acid. **b** Structural view highlighting residues experiencing paramagnetic relaxation enhancement quantified by the intensity ratio (*I*_para_/*I*_dia_) with *I*_para_/*I*_dia_ = 1 indicating no effect of the spin label. The ratio is indicated by gradual coloring from red (*I*_para_/*I*_dia_ = 0) to white (*I*_para_/*I*_dia_ = 1). Black parts indicate residues with missing assignments or severe peak overlap. **c** The intensity ratio (*I*_para_/*I*_dia_) shown per residue. Residues for which quantification was impeded by missing assignments or peak overlap are red and grey, respectively, on the x-axis. G188, which is the site of MTSL labeling is indicated by orange spheres or an asterisk in **b** and **c**, respectively

Recent work has shown that deleting all 15 residues of the NCS-1 C-terminal leads to a destabilization of the N-domain by 15.4 kJ mol^−1^, without affecting the C-domain, although no specific contacts between the tail and the N-domain were established [33]. Structural studies of NCS-1 alone by NMR [33] and molecular dynamics (MD) simulations [38], and in complex with PIK1 by X-ray crystallography [42], show that the position of the C-terminal tail varies. To address this conundrum and determine potential dynamic contacts that the tail makes across the protein, we employed site-directed paramagnetic spin labeling in the C-terminal tail of NCS-1 (Fig. 2, S1). Paramagnetic relaxation enhancements (PREs) were quantified from resonance intensity-ratios obtained from BEST-TROSY spectra recorded in paramagnetic and diamagnetic states (Fig. 2a). Attachment of the *S*-(1-oxyl-2,2,5,5-tetramethyl-2,5-dihydro-1H-pyrrol-3-yl)methyl methanesulfonothioate (MTSL) label at position 188 (involving two mutations: C38S and G188C) in the C-terminal tail caused substantial line broadening mainly of resonances belonging to residues in the N-domain, supporting an interaction between the tail and the N-domain (Fig. 2b,c). In addition, line broadening was observed in H6 and H7 from EF3 for residues in the vicinity of the hydrophobic cleft that forms the binding site of PIK1. All missing peaks were recovered upon reduction by addition of excess ascorbic acid, attesting to the lack of structural perturbation by the label (Fig. 2a, S1). These observations support previous suggestions that the tail is not static but dynamically contacts many sites in NCS-1 [33]. The contacts we identify are in good agreement with those proposed by Bellucci et al*.* in their study of NCS-1 using MD simulations [38]. However, it is not clear from their data or ours, if any of these contacts require specific residues within the tail or if it is fluctuating dynamically to allow for an efficient exchange by incoming ligands.

**Fig. 3:**
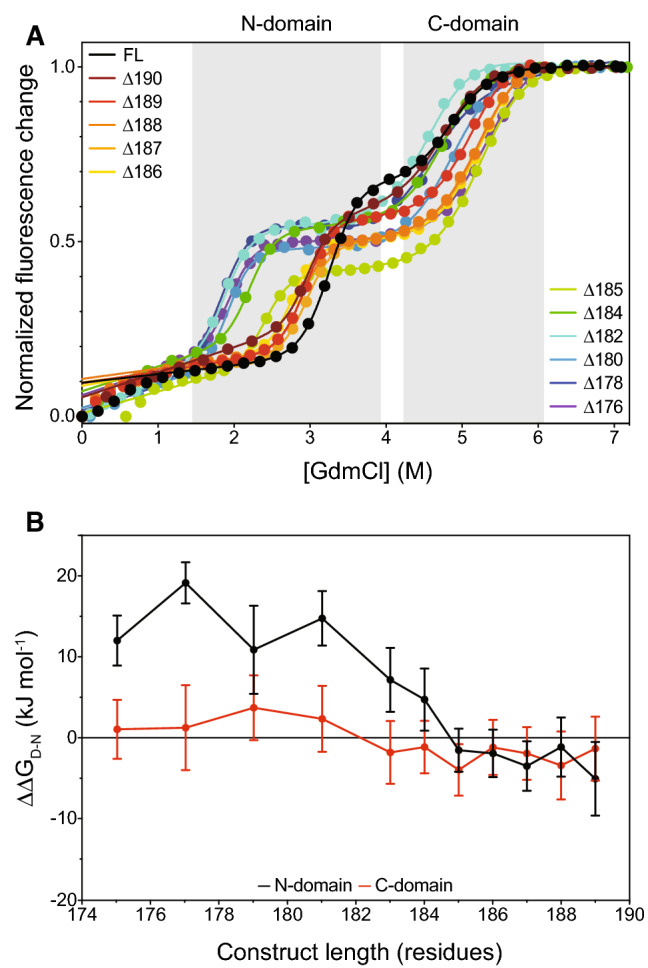
Thermodynamic comparison of NCS-1 wild type and truncation variants from equilibrium unfolding studies measured by intrinsic fluorescence. **a** Equilibrium unfolding curves measured by average emission wavelength as a function of denaturant concentration. **b** Plot showing the dependence of ${\Delta \Delta G}_{D-N}^{{H}_{2}O}$ on construct length

To address this, and identify whether there are any crucial residues, and to establish how much of the tail is required for the stabilizing effect [33], we designed and produced 11 truncated variants, sequentially deleting one or two residues at a time from the C-terminus. The thermodynamic behavior of these NCS-1 variants in guanidinium chloride (GdmCl) was compared for all truncations against the full-length (FL) protein (Fig. 3, S2A,B, Table 1). All variants maintained a 3-state unfolding reaction as described earlier for the FL variant [37, 45, 46], with an intermediate state consisting of an unfolded N-domain and folded C-domain (Fig. 3a) [36]. For many of the variants, we observed a non-linear increase in fluorescence emission at low concentrations of GdmCl, suggestive of a potential third transition. However, no corresponding effects on helicity were inferred from CD spectroscopy (Fig. S2C). Furthermore, NMR titrations using up to 1 M GdmCl resulted in chemical shift perturbations that were largest for charged residues throughout the protein (Fig. S2D,E). These perturbations could be fitted to a binding model with an average *K*_d_ of ~ 380 mM (Fig. S2F,G), which is in a similar range as previous observations of GdmCl binding [47]. Thus, it is more likely that the change in fluorescence emission at low denaturant concentrations is related to weak binding of GdmCl, rather than structural perturbations. Deletion of residues 186–190 had no substantial effect on stability, however, after deletion of residue Leu185, the N-domain was increasingly destabilized for each additional residue removed (Fig. 3b, Table 1). The largest destabilizing effects were seen after deletion of Ala182 reaching a plateau with a destabilization effect of ~ 14 kJ mol^−1^, suggesting residues 183–185 to be of importance. In contrast, the C-domain appeared largely unaffected by the tail truncation, maintaining a ${\Delta G}_{D-N}^{{H}_{2}O}$ of ~ 42 kJ mol^−1^ for all variants. Due to large errors associated with the *m*-values, it was difficult to judge if the compactness of the domains was affected, and the ratios of the amplitudes for the N- and C-domain transitions were, therefore, plotted against protein length to see if this suggested a large change in the environment surrounding the tryptophan residues (Fig. S2H). These ratios did not follow a distinct pattern based on the number of residues removed and were all within error of the FL value, suggesting the relative compactness of the domains to be maintained.

**Table 1:** Stability of NCS-1 truncates in GdmCl

Protein	Terminal residue	Overall charge	${\Delta \mathrm{G}}_{D-N}^{{H}_{2}O}$ (kJ mol^−1^) N-domain	${\Delta \mathrm{G}}_{D-N}^{{H}_{2}O}$ (kJ mol^−1^) C-domain	${\Delta \Delta \mathrm{G}}_{D-N}^{{H}_{2}O}$ (kJ mol^−1^) N-domain	${\Delta \Delta \mathrm{G}}_{D-N}^{{H}_{2}O}$ (kJ mol^−1^) C-domain
Wild type	Val190	− 9	42.0 ± 1.9	42.0 ± 2.8	–	–
Δ190	Leu189	− 9	47.0 ± 4.1	43.4 ± 2.8	− 5.1 ± 4.6	− 1.3 ± 4.0
Δ189	Gly188	− 9	43.1 ± 3.1	45.4 ± 3.1	− 1.4 ± 3.6	− 3.4 ± 4.2
Δ188	Asp187	− 9	45.4 ± 2.4	43.9 ± 1.7	− 3.5 ± 3.1	− 1.9 ± 3.3
Δ187	Tyr186	− 8	43.9 ± 2.2	43.2 ± 1.9	− 1.9 ± 3.0	− 1.2 ± 3.4
Δ186	Leu185	− 8	43.5 ± 1.8	46.0 ± 1.4	− 1.5 ± 2.7	− 3.0 ± 3.2
Δ185	Ser184	− 8	37.2 ± 3.3	43.2 ± 1.6	4.8 ± 3.8	− 1.4 ± 3.2
Δ184	Leu183	− 8	34.8 ± 3.4	43.8 ± 2.7	7.2 ± 3.9	− 1.8 ± 3.9
Δ182	Gln181	− 8	27.2 ± 2.8	39.7 ± 3.0	14.8 ± 3.4	2.3 ± 4.1
Δ180	Ile179	− 8	31.1 ± 5.1	38.3 ± 2.8	10.9 ± 5.5	3.7 ± 4.0
Δ178	Pro177	− 8	22.8 ± 1.7	40.8 ± 4.4	19.1 ± 2.6	1.2 ± 5.2
Δ176	Ala175	− 7	29.9 ± 2.4	41.0 ± 2.3	12.0 ± 3.1	1.1 ± 3.6

**Fig. 4:**
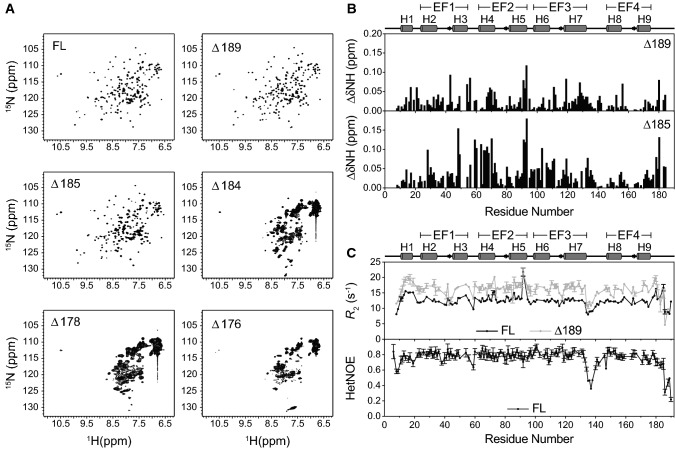
Comparison of NCS-1 truncates by NMR. **a** ^1^H,^15^N-HSQC spectra of NCS-1 FL, Δ189, Δ185, Δ184, Δ178 and Δ176. **b** Chemical shift perturbations upon truncation of NCS-1 in Δ189 and Δ185. **C** Top shows transverse relaxation rates (*R*_*2*_) of NCS-1 FL and Δ189. Bottom shows ^1^H,^15^ N heteronuclear NOEs of FL NCS-1

The overall dynamic properties of the NCS-1 truncation variants were next assessed using NMR spectroscopy. Previous NMR studies of NCS-1 including its structure determination have been performed at 37 ℃ [33], whereas folding experiments using single-molecule optical tweezers have been performed at 25 ℃ [35]. A temperature titration of FL NCS-1 from 25–37 ℃ showed no signs of structural perturbations and we therefore continued our NMR analyses at 37 ℃ for comparative purposes (Fig. S3). Again, removal of the first five residues had no distinct impact on the quality of the ^1^H,^15^N-HSQCs until removal of Leu185 (Fig. 4a), after which distinct line-broadening and loss of peaks consistent with strongly altered dynamics were seen (Fig. 4a). As the thermodynamic behavior did not indicate a presence of unfolded protein (Fig. 3a), we asked if the severe line broadening in the NMR spectra could instead be caused by the formation of oligomeric species. The NMR samples were run on analytical size-exclusion chromatography and no oligomeric species were observed at protein concentrations of 1–10 and 100–150 µM corresponding to the concentrations used for fluorescence and NMR experiments, respectively, (Fig. S4). However, in attempts to analyze the protein by small angle X-ray scattering we did observe aggregation of some truncated variants at these elevated concentrations, suggesting an increased hydrophobic exposure. To substantiate this, ANS binding to FL NCS-1 and variants was analyzed, revealing a much more intense ANS fluorescence being most pronounced in the Δ180 variant (Fig. S5). Thus, the severe line broadening is not due to aggregation, but rather introduction of dynamics on a timescale that is unfavorable for NMR measurements. We, therefore, compared the transverse relaxation rates (*R*_2_) and chemical shift perturbations for NCS-1 FL, Δ189 and Δ185 (Fig. 4b, c). While there were subtle chemical shift perturbations that increased in size with the degree of truncation, larger differences were seen in the relaxation rates. For Δ189, we observed a substantial increase in average *R*_2_ rates (~ 30%) throughout most of the protein, likely caused by contributions from chemical exchange (Fig. 4c). We furthermore attempted measurements of *R*_2_ rates for the Δ185 variant, however, they were too fast for a reliable determination using the range of relaxation delays investigated. Besides the neighboring residues, the CSPs experienced for both Δ189 and Δ185 located mainly to the N-domain and EF3 of the C-domain (Fig. 4b). EF3 is part of the interface between the N- and C-domains and makes numerous contacts to EF2 of the N-domain (Fig. 1a). In contrast, EF4, which is more distant to the N-domain, experienced only minor CSPs in accordance with the PRE-data. The massive changes in the NMR spectra for truncations beyond residue 185 is in good agreement with the tipping point for the destabilization observed from the fluorescence data. In further agreement, backbone dynamics analyzed in ^1^H,^15^N heteronuclear NOE experiments revealed that residues 176–185 of the tail have less flexibility than the reminder of the tail with an average NOE value of ~ 0.72, whereas the remaining tail residues appear highly disordered (average NOE value of ~ 0.37) (Fig. 4c).

To summarize, the disordered C-terminal tail effectively stabilizes the N-domain, where residues 183–185 represent a crucial segment for both thermodynamic stability and the dynamical signature throughout the entire protein sequence.

### Is sequence important, or just disorder?

**Fig. 5:**
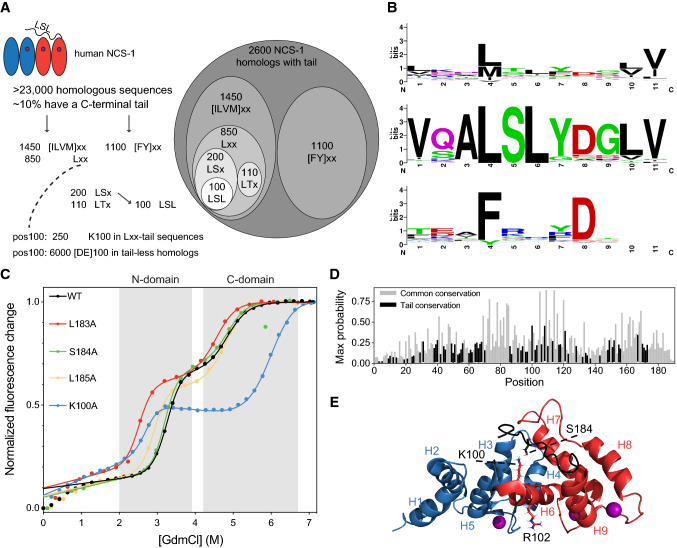
NCS-1 conservation and structure. **a** Analysis of tail sequence patterns in NCS-1 homologs. Only 10% of the NCS-1 homologs identified by HHblits have tails (details see Methods). **b** Logos showing sequence conservation of NCS-1 homologues having a C-terminal tail. Top and bottom plots show homologues with either an aliphatic residue or aromatic residue at position 183, respectively. The middle plot shows homologues that align with LSL at positions 183–185. **c** Chemical stability of variants L183A (red), S184A (green), L185A (yellow) and K100A (blue). **d** Conservation analysis of tail homologues compared to all homologues. The most conserved positions common to NCS-1 homologous regardless of a tail (gray) and common to NCS-1 homologues with tail (black) with K100 being the most conserved. **e** Visualization of the interaction between K100 and S184 (sticks) in the C-terminal tail, based on PDB 2lcp [33] and rendered using PyMol

The key tail-residues in NCS-1 as identified by truncations are residues L183, S184 and L185 (Figs. 3 and 4). We, therefore, asked if these residues formed a conserved motif across the family. Based on the NCS-1 sequence, we identified 23,000 homologous sequences. Multiple sequence alignment, anchored in the Ca^2+^-binding sites, revealed that about 10% of the homologs (2600) had a C-terminal tail (Fig. 5a, S6 and Methods). Of these, 1450 had [ILVM] at position 183 (Fig. 5a, b top). Ser and Thr were the most common residues at position 184 (~ 200 and ~ 110 occurrences, respectively), suggesting a potential need for a hydrogen bond donor. Position 185 was either [AVILM] in 774 sequences (30%) or aromatic in 223 sequences (8%). Around 100 sequences contained the complete LSL motif, with high sequence conservation in the entire tail (Fig. 5b, middle). Another large branch of the tail sequence − space constituting ~ 1100 sequences contained aromatic side chains (predominantly Phe) at position 183 (Fig. 5b bottom). These typically had large charged or polar residues in the following positions (Arg, Gln, Lys) as well as a negatively charged residue at the position corresponding to 187. Thus, sequence analyses pointed towards an aliphatic-S/T-aliphatic motif for the NCS-1-like subfamily. While there is considerable sequence variation in the key residues, aliphatic residues are preferred at position 183. Position 184 is less constrained and may depend on context. Position 185 is the least constrained but is often hydrophobic. Some tails had other preferences, likely testifying to different binding pockets or different interactomes.

To substantiate the role of these specific residues, we substituted each of them for alanine in FL NCS-1 and compared their stabilities to that of the wild-type (Fig. 5c). The precision of the m-value determination made a quantitative comparison complicated (Table 2), but qualitatively, S184 was not required for the tail-stabilizing effect implying that particular hydrogen bonding is less important. In contrast, the L183A and L185A mutations both destabilized the N-domain, with the largest effect seen for L183 (Fig. 5c). The C-domain was largely unaffected by the mutations. We next asked if L183-S184-L185 would make specific contacts to residues in the N-domain. We calculated the conservation of the most common residue at each position and partitioned the homologous sequences into those with a C-terminal tail and those without. The most conserved positions are common to all NCS-1 homologues regardless of whether a tail is present (Fig. 5d, gray bars). Among the subset specific to NCS-1 homologues with a tail, position 100 is most conserved, followed by positions 108 and 121 (Fig. 5d, black bars). In the structure of NCS-1, these three residues point into the binding cleft in the C-domain. K100 is particularly interesting as it borders the two domains, is *vis-a-vis* to a disease-causing mutation R102Q linked to autistic spectrum disorder, and in the NMR ensemble it is very close to S184 (Fig. 5e). A K100A substitution had a dramatic effect on the stability of both domains, not only destabilizing the N-domain by 2.0 ± 3.9 kJ mol^−1^, but also substantially increasing the stability of the C-domain by − 4.8 ± 3.7 kcal mol^−1^ (Fig. 5c, Table 2). Notably, variants without a tail prefer Glu at position 100 (Fig. 5a). These observations further point toward a role of position 100 in tail-communication as well as in function. We also assayed and compared the new variants using the ANS-binding assay and by NMR (Fig. S5). All variants had a nicely dispersed ^1^H,^15^N-HSQC spectrum, but compared to wild type, the Leu to Ala variants had an increase in ANS fluorescence intensity, indicating more exposed hydrophobicity. In fact, their fluorescence intensities were comparable to that of Δ180 where most of the tail is missing (Fig. S5). Again, the S184A variant was indistinguishable from wild type, and the K100A showed a decrease in ANS fluorescence intensity suggesting that this substitution increases the residence-time of the tail within the groove, shielding the hydrophobic core and the binding site. In contrast, the R102Q variant had an increased ANS fluorescence, suggesting that the tail, which is less dynamic in the R102Q variant [33], provides access to the binding site in this variant. Of relevance, the most perturbed chemical shifts of the R102Q variant were V180 and L183 [33].

**Table 2:** Thermodynamics of variants and tail swaps

Protein	${\Delta \Delta \mathrm{G}}_{D-N}^{{H}_{2}O}$(kJ mol^−1^) N-domain	${\Delta \Delta \mathrm{G}}_{D-N}^{{H}_{2}O}$(kJ mol^−1^) C-domain
K100A	2.0 ± 3.9	− 4.8 ± 3.7
L183A	5.2 ± 5.9	− 1.6 ± 5.1
S184A	− 6.7 ± 3.7	− 6.9 ± 7.0
L185A	− 1.1 ± 3.8	− 1.1 ± 4.6
KChIP2	− 4.4 ± 2.5	− 8.7 ± 3.8
cNCS-1	8.0 ± 3.1	1.6 ± 3.6
Recoverin	2.6 ± 4.7	− 9.7 ± 4.1

Together, these data suggest that sequence properties of the tail, and in particular the LSL motif L183-S184-L185, is key for tail communication and with positional preferences across the tail-carrying calcium sensors involving a degree of hydrophobicity. It also suggests that the regions surrounding K100, including R102, have an important—though so far enigmatic—effect on stability of both domains, and may be optimized for function rather than stability.

### Will a tail from any calcium sensor do?

**Fig. 6:**
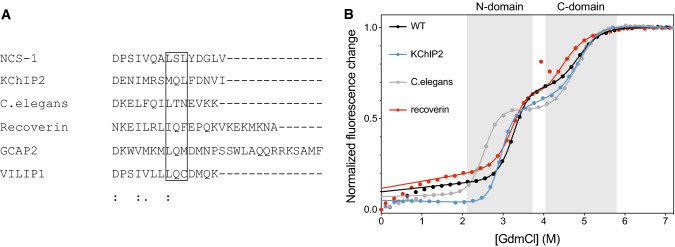
Tail-swap variants. **a** Sequence alignment of tails, highlighting possible conserved positions. **b** Equilibrium unfolding curves for the KChIP2 (blue), *C.elegans* (gray) and recoverin (red) swap variants compared to WT NCS-1 (black)

Generally, and made possible by its disordered nature, the sequence of the tail is not particularly conserved among calcium sensors and many tails do not have the same key residues as NCS-1. This lack of conservation is often observed for disordered regions, and not in disagreement with a key biological function. Several homologues have tails of variable composition and length, alluding to various different properties. To evaluate if any of these flexible tails could stabilize NCS-1, we designed a series of tail-swaps from some of the main evolutionary groups [18]. C-terminal tails from the potassium channel interacting protein 2 (KChIP2), recoverin and NCS-1 from *C. elegans* (cNCS-1) were chosen as they represent different lengths and sequence properties (Fig. 6a, Table S2). The tail from KChIP2 is the same length as human NCS-1 and has the same net charge of − 2, but the number of charged residues is higher. Recoverin has a tail that is seven residues longer and has an overall charge of + 3. The cNCS-1 tail is one residue shorter and has no overall charge despite having almost four times as many charged residues. At position 183, cNCS-1 has a Leu, recoverin an Ile and KChIP2 a Met, whereas Ser184 is replaced by Gln in recoverin and KChIP2, and Thr in cNCS-1. Leu185 is conserved in KChIP2, but is replaced by Phe in recoverin, and Asn in cNCS-1*.*

We compared the stability of the tail-swaps in GdmCl (Fig. 6b). All three chimera proteins were stable, and displayed 3-state behavior similar to wild type NCS-1 [33]. Unlike the truncations, there were only minor effects from changing the tail sequence; both the recoverin and KChIP2 swaps had little effect on either domain (Table 2). In contrast, the cNCS-1 tail swap destabilized the N-domain. Whether this was caused by a lack of hydrophobicity at position 185, the presence of a highly positively charged C-terminus, or other traits, remains to be addressed. Remarkably, none of the chosen tails destabilized the N-domain to a similar extent as the full tail-deletion. Instead, they were excellent mimics of the native tail. In accordance with this conclusion, the NMR spectra of the chimeras revealed nicely resolved NMR spectra with little change in dynamic behavior (Fig. S7). Given the dynamic interplay of the tail with a large part of NCS-1, the majority of peaks were expectedly shifted with only 7–10% peaks overlapping, making identification from the wild-type assignment difficult. Still, all tails conferred properties that rescued the dynamics and maintained contacts broadly to the entire NCS-1.

Overall, the tail swap results indicate that besides the loose hydrophobic motif at positions 183–185, there is no other strong sequence coupling between the two ordered domains and the tail. It appears that hydrophobic tail residues existing within a dynamic context are necessary to dynamically shield the hydrophobic binding cavity and thereby stabilize the N- and C-domains.

### The disordered tail controls kinetics of ligand binding

**Fig. 7:**
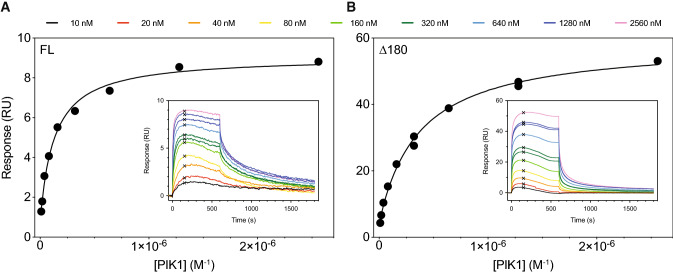
Functional impact of the C-terminal disordered tail. Binding isotherms for PIK1_121-172_ in solution binding to immobilized FL NCS1 (**a**) and Δ180 (**b**) measured by SPR at 25 °C. The binding isotherms were constructed from the binding response (RU) at 150 s after sample injection (best approximation to steady-state conditions, marked by black cross). The recorded sensorgrams for multicycle analysis of twofold dilution series of PIK1_121-172_ (10 nM–2.6 µM) are shown as insets in panels **a** and **b**

To address the functional impacts of the C-terminal disordered tail of NCS-1, we measured the real-time binding kinetics between FL NCS-1 or Δ180 and a 52-residue synthetic fragment of the well-established partner PIK1 using surface plasmon resonance (SPR). Under steady-state conditions, we find that PIK1_121-172_ bound immobilized FL NCS-1 and Δ180 with slightly different affinities at 25 °C with *K*_d_-values of 130 ± 21 nM (FL) and 364 ± 40 nM (Δ180), respectively (Fig. 7a,b). Notably, visual examination of the binding profiles at non-equilibrium suggested that the *K*_d_-values were predominantly driven by differences in the dissociation rate constants (*k*_off_). Analyses with multi- and single-cycle protocols were therefore used to calculate association (*k*_on_) and dissociation (*k*_off_) rate constants. Although the kinetics of PIK1_121-172_ binding to neither FL NCS-1 nor Δ180 complied well to a simple 1:1 binding reaction and yielded suboptimal fits, which suggested a more complicated binding mechanism, we nonetheless made a rough estimate on the rate constants using local fitting of the initial phases of the dissociation phase (Table 3). We found the complex with FL NCS-1 was longer-lived compared to Δ180, with *k*_off_ 4.3 × 10^−3^ s^−1^ and *k*_off_ 25 × 10^−3^ s^−1^, respectively. This may reflect the difference in the inherent stability of these proteins and show that the disordered tail may indirectly control the lifetime of the complex.

**Table 3:** Kinetics of NCS-1:PIK1 interaction

Protein	*k*_on_ (10^5^ M^–1^ s^–1^)	*k*_off_ (10^–3^ s^–1^)	*K*_D_ (nM)	*R*_max_	*K*_D, EQ_ (nM)
FL NCS-1	2.0 ± 1.1	4.3 ± 0.6	29 ± 21	8.2	130 ± 21
Δ180	2.4 ± 2.2	25.1 ± 0.3	219 ± 211	55	364 ± 40

## Discussion

### Both specific and non-specific interactions contribute to the stabilizing effect of the tail

Based on truncations, substitutions and tail swaps, our data suggest that the contacts made by the tail are important for the stability of the N-domain and for the dynamics of both domains. Mainly, the contacts are dynamic and non-specific and contribute to stability largely by being hydrophobic. An aberrant tail, albeit still harboring specific chemical characteristics at key positions, was better than no tail at all for the N-domain stability. Yet, if the dynamic behavior was solely dependent on specific contacts, the HSQC spectra would not be as well resolved as observed, rather the spectra for the tail swaps would look like those of the truncates Δ176–Δ184. Thus, the substituted tails must be making stabilizing contacts with the binding cleft of NCS-1 in a similar dynamic way as the endogenous tail. Still, analysis of subfamily members with tails shows that tail sequences overall have low conservation, consistent with our tail swap experiments showing that no strong sequence dependence was encoded in this region. Therefore, individual tails may well have evolved to fine-tune the stability of each protein, i.e. by tuning the time of the tail spent in the cavity. In this way, the tail would control ligand exchange as suggested from earlier work [43, 44] and shown here for NCS-1 where tail-effects affected the lifetime of the complex, likely indirectly through modulation of protein stability. The tail lacking one of the critical hydrophobic residues of the motif, from *C. elegans*, was the only one imposing substantial destabilization. The common denominator of the tails is their disordered nature (Table S2). If the tail was ordered, tolerance of variation would likely be lower, and the fine-tuning seems to be possible because of the pronounced dynamics, which instead of giving the dynamics an *on–off* character, allow for a continuum of global dynamics that are controlled by the disordered tail [48]. The recoverin tail is very different and still has about the same effect on stability as the wild-type, re-enforcing the idea that tail dynamics are critical. Nevertheless, as our mutational experiments reveal, the sequence has an influence on tail functionality, although not as pronounced as seen for MutL*α* (Mlh1-Pms1) mismatch repair (MMR) complex, where scrambling ablated function [49], but similar to the tail-function in CIB1, where the tail was displaced upon ligand binding [44]. Finally, it is possible that the tail of NCS-1 may also affect Ca^2+^-binding, as observed for a calmodulin variant from rice [43], but this remains to be addressed.

### A dynamic ligand mimic that lacks folding-upon-binding properties

**Fig. 8:**
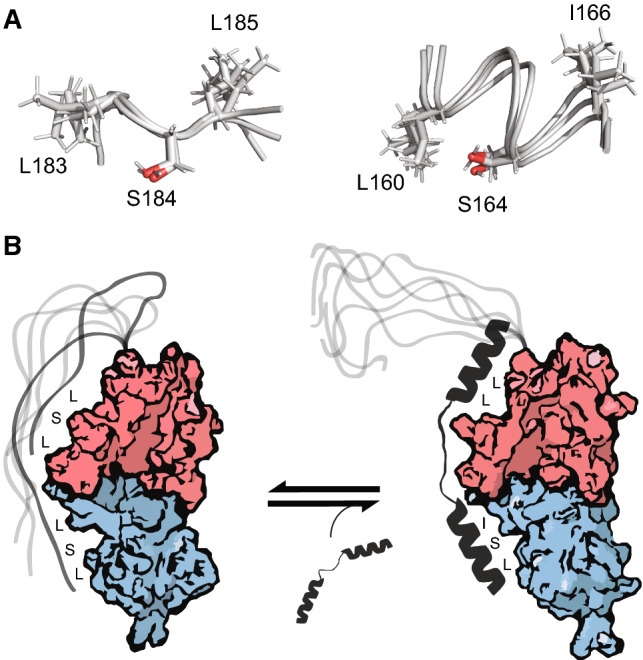
Ligand mimic behavior of the NCS-1 C-terminal disordered tail. **a** Orientation of the LSL-motif in the disordered tail of NCS-1 (left) and the orientation of the LX_3_SXI motif in the binding helix of the NCS-1 binding partner PIK1 (right). **c** Illustration of how the LSL motifs dynamically occupy the binding sites of NCS-1 without folding into the pockets and how this is displaced by the ligand upon binding

Previous work on NCS-1 in Drosophila has shown that residues Leu183, Ser184 and Leu185 (L182, S183 and L184 in Drosophila) play a role in partner recognition, acting as one side of a “hydrophobic cage” within the binding cleft [27]. To assess whether this is dependent on the binding partners, and therefore specific to different calcium sensors, we looked at the structure of NCS-1 in complex with the binding partner PIK1 [42] to determine if Leu183, Ser184 or Leu185 were key contacts in multiple interactions. PIK1 binds to the hydrophobic cleft of NCS-1 forming an α-helix in each domain cleft that presents a Leu, a Ser, and an Ile in a very similar orientation to that observed by the C-terminal tail in some of the NMR models in the ensemble (Fig. 8a) [33]. In the free proteins, the LSL-motif of the tail is presented in an extended chain conformation, which roughly equates in space to the LX_3_SXI of PIK1, structured in folded helices when bound (Fig. 8a). Similar motifs, also in folded α-helices, are seen in the other known binding partners of NCS-1, dopamine D2 receptors B and C (FX_2_AF) and the GPCR kinase GRK1 (VX_3_AF) [50]. It is worth noting that PIK1 at the end of the helix bound in the N-domain, presents a serine that forms a hydrogen bond to the side chain of K100. Thus, it is possible that the -LSL- residues of the tail act as a short “motif” fitted to match the binding cleft, primarily in the N-domain where we see most effects, but likely also in the C-domain, as seen from the MTSL and chemical shift effects observed around residues 120–140. Thus, the tail functions as a general ligand mimic of the NCS-1 binding partners (Fig. 8b). Since the motif of the tail is disordered, it lacks the coupled folding and binding energy, which is likely harvested by the partner proteins when forming a helical structure. Indeed, in binding studies comparing NCS-1 and NCS-1 without the tail, a pronounced effect on affinity and especially the lifetime of the complex was seen (Fig. 7a, b). Thus, the tails have evolved to never become true competitors. Furthermore, once the partner helix binds, further stabilization of the EF hand core structures are possible, leading to allosteric conformational changes important for function. How binding of different partners to NCS-1 affects its stability and overall fold is not currently deductible from available structures and will have to await further studies.

### Why does order need disorder?

Out of more than 20,000 homologous sequences of neuronal calcium binding proteins, only 11% have tails. What are the benefits of having a disordered tail? There is very little functional redundancy within the NCS subfamily, which perhaps may be gauged by the presence of a disordered tail. As mentioned, the binding cleft of these proteins can be blocked by an intrinsic feature such as the N-terminal myristoyl group of recoverin, and the C-terminal tail of NCS-1. It is possible that this is done by tuning of the binding kinetics via control of the dynamic motions that define how effectively a ligand can bind. From our results, it is clear that the stability and structure of NCS-1 rely on its tail. Thus, without this stabilizing force the protein would have a looser 3D structure, which may facilitate a wider—or different—range of protein–protein interactions to form, or lead to its degradation. Such a scenario is also facilitated by modulating the ions bound to the proteins, as the Mg^2+^-bound state and the apo state of NCS-1 are both destabilized [51].

Many eukaryotic and particularly mammalian proteins have long disordered linkers or tails flanking ordered domains [8]. Given our findings that the disordered tail impacts stability of the globular domains, and have ligand-mimicking properties, we speculate that the C-terminal tail of NCS-1, and disordered linkers in general, may allow fast and dynamic regulation of thermodynamic properties of the protein, e.g. to promote or reduce interactions with ligands or other proteins. This is the case e.g. for the disordered N-terminal of Src kinases, which has multiple weak interaction with the folded domain of yet incompletely understood function [48], and for the glucocorticoid receptor, which dynamically regulates transcription via cross-talk between disordered and ordered segments [52].

A number of bioinformatics studies have analyzed disordered elements from a proteome-wide perspective, however such an ‘omics’ view cannot directly inform us on the molecular interplay between order and disorder. Although completely disordered complexes exist [53], IDP interactions have mostly been discussed in the context of short linear motifs (SLiMs) [54] and indeed, intermolecular interactions between SLiMs and ordered domains are perhaps the most established case of intermolecular communication between ordered and disordered elements. In an artificial system of a folded, stable protein (GFP) with fused intrinsically disordered tails, changes to overall protein conformation in the fusions were observed [14]. Although only a small number of fusions were tested, no clear patterns could be extracted indicating that the interplay between order and disorder is likely complex and context dependent [56]. But where a synthetic system can help uncover biophysical effects, these will not have been tuned by evolution. A naturally evolved system of order and disorder such as the one studied here in the form of NCS-1, or one of the many existing in modular proteins, is likely better suited for studying the functional interplay of these elements. Here evolution has optimized the biological function of the protein in its native context by tuning and gearing these intramolecular interactions between order and disorder.

## Conclusion

In this study, we have shown that both the length and the sequence of the C-terminal tail of NCS-1 contribute to the overall (thermo)-dynamic behavior of the globular domains of the protein. When a threshold number of residues are removed from the C-terminus, the tail no longer reaches its vital contacts in the N-domain. However, the most important contacts the tail makes require specific chemical properties. In at least one binding partner [42] the apparent -LSL/I- motif is present, and similar motifs can be found in other ligands, where they are presented in helical contexts. Thus, the disordered tail of NCS-1 acts as a dynamic ligand mimic with fit-in motifs of recognition to both domains and controls the kinetics of interaction. Given the abundance of disordered regions in eukaryotic proteins, understanding how these influence folded domains, and vice versa, remains critical.

## Materials and methods

### Protein expression and sample preparation

The NCS-1 variants were generated using the Quikchange (Agilent) site-directed mutagenesis kit and primers obtained from TAG Copenhagen. NCS-1 and variants were expressed without the myristoyl group. Expression and purification was done as previously described [33, 37]. For MTSL labeling, protein samples (~ 1 mg/mL) were reduced in a buffer consisting of 100 mM Tris HCl, pH 7.0 and 2 mM dithiothreitol (DTT) overnight at room temperature. DTT was removed by dialysis overnight, while nitrogen was bubbled through the sample to remove oxygen. 6 M guanidinium chloride (GdmCl) and a tenfold molar excess of MTSL was added, and the pH of the sample was raised to 8.0 with NaOH. The labeling reaction was allowed to proceed for 16 h at room temperature. Unlabeled and labeled proteins were separated by reversed-phase HPLC, using a Zorbax C18SB 17 mL column and a gradient of buffer A (MilliQ, 0.1% trifluoroacetic acid (TFA)) and buffer B (100% acetonitrile, 0.1% TFA) over seven column volumes (CVs) at 2 mL/min. Labeling was confirmed by an observed mass increase of ~ 185 Da by MALDI-TOF mass spectrometry after the last HPLC purification step.

### Size-exclusion measurements

Samples of NCS-1 Δ184 at concentrations of 10, 50, 100, 200, and 540 μM were analyzed on an analytical Superdex 75 10/300 GL column equilibrated with 2 CVs of 20 mM Tris HCl, 180 mM NaCl, 5 mM CaCl_2_, 5 mM DTT, pH 7.2, at room temperature. The samples were applied at a flowrate of 0.5 ml min^−1^ and eluted over 1.5 CVs in fractions of 0.5 ml. The column was run on an ÄKTA HPLC instrument and all buffers were filtered (0.45 μM filter) before use. The eluted fractions were analyzed by SDS-PAGE.

### Stability measurements

Equilibrium experiments were done using a PerkinElmer Fluorimeter or Jasco J-810 Spectropolarimeter at 25 ℃ in 10 mM Tris, 5 mM DTT, 5 mM CaCl_2_, pH 7.2 at increasing concentration of GdmCl, determined by measuring refractive index. The final protein concentration was 1–5 µM for fluorescence and 10 µM for CD. Since the calcium concentration determines which of the domains are observed by these methods [37] all equilibrium curves were measured at the relatively high concentration of 5 mM CaCl_2_ so that both transitions could be distinguished. Fluorescence experiments were performed with excitation at 295 nm, while monitoring emission in the range from 300–400 nm. Emission spectra were averaged over three scans and the average emission wavelength (< *λ* >) was calculated according to:

1 $$< \lambda > = \frac{{\sum\nolimits_{i = 1}^{N} {F_{i} \lambda_{i} } }}{{\sum\nolimits_{i = 1}^{N} {F_{i} } }}$$

For CD the change in helical structure was followed from the absorption in the range from 220–224 nm. Spectra were recorded with a bandwidth of 2 nm, response time of 0.25 s and a scanning speed of 5 nm/min. Eight accumulations were recorded and averaged for each point. The raw CD signal was then averaged from 220–224 nm.

The fraction of folded protein was calculated from the maximum and minimum < *λ* > for fluorescence or raw CD signal for CD, and plotted against the concentration of denaturant. All curves were fitted to a 3-state unfolding equation:

2 $$\begin{aligned}f\left(x\right)&=\left({a}_{0}+{a}_{1}\left[\mathrm{C}\right]\right)+\left({a}_{2}+{a}_{3}\left[\mathrm{C}\right]\right){e}^{\frac{{m}_{1}\left(x-C{\mathrm{m}}_{1}\right)}{RT}} +\left({a}_{2}+{a}_{3}\left[\mathrm{C}\right]\right)+\left({a}_{4}+{a}_{5}\left[\mathrm{C}\right]\right){e}^{\frac{{m}_{2}\left(x-C{\mathrm{m}}_{2}\right)}{RT}} \end{aligned}$$

where [C] is the GdmCl concentration and *a*_0_, *a*_1_, *a*_2_, *a*_3_, *a*_4_, *a*_5_ are slopes and intercepts of the linear dependencies of the different states. Where possible unfolding and refolding curves were fitted globally. The conformational stability of N- and C-domains of each NCS-1 variant was calculated as follows:

3 $$\Delta {G}_{D-N}^{{\mathrm{H}}_{2}\mathrm{O}}=m{ C}_{\mathrm{m}.}$$

and the change in free energy of unfolding was then determined from analysis of the denaturation data as follows:

4 $${\Delta \Delta G}_{D-N}={\Delta G}_{D-N}^{WT}- {\Delta G}_{D-N}^{mut}$$

### ANS-binding assay

Fluorescence spectra were recorded on a PerkinElmer Fluorimeter at 25 ℃ in 10 mM Tris 5 mM CaCl_2_, 5 mM DTT, (pH 7.2). The protein concentration was 10 µM and ANS was added in 10 × excess at 100 µM. The samples were excited at 370 nm and emission followed from 400 to 600 nm, averaged over ten scans with a scan rate of 50 nm min^−1^ and slit widths of 5 nm.

### NMR

All experiments were carried out at 37 ℃ in 10% (v/v) D_2_O, 10 mM Tris, 5 mM CaCl_2_, 2 mM MgCl_2_, 5 mM TCEP, 0.02% (w/v) NaN_3,_ pH 7.2. Protein concentrations ranged between 100 and 150 µM. The experiments were recorded on a Bruker AVANCE spectrometer operating at 600 MHz and equipped with a cryoprobe. All spectra were processed using NMRPipe or Topspin (Bruker) and ^1^H chemical shifts were referenced to the methyl proton resonances of 2,2-dimethyl-2-silapentane-5-sulfonic acid (DSS) at 0.0 ppm. Assignments for NCS-1 were taken from BioMagResBank, accession number 4378 [34]. For NCS-1_C38S G188C_ and the truncation variants, resonance assignment was performed using a ‘minimum chemical shift’-like procedure [56].

Experiments for determining the *R*_2_ relaxation rates and ^1^H,^15^N Heteronuclear NOEs of FL NCS-1 and the Δ189 variant were recorded on samples containing ^15^N-labeled protein. For *R*_2_ relaxation, the delays were 17 ms, 3 × 34 ms for error estimation, 68, 102, 3 × 136, 170, 204 and 237 ms and a recycle delay of 2.5 s were used in all experiments. The relaxation decays were fitted to a single exponential function and the relaxation times determined using the CcpNmr Analysis software [57]. For NOEs, spectra were recorded with and without proton pre-saturation and a recycle delay of 5 s.

For chemical shift perturbation (CSP) analysis, ^1^H-^15^ N-HSQC spectra of ^15^N-labeled NCS-1 and variants were recorded and analyzed with CSPs calculated as combined amide chemical shift changes using the following formula [58]:

5 $${\Delta \delta }_{NH}\left(ppm\right)=\sqrt{(\Delta {\delta }^{1}H{)}^{2}+(0.154 {\Delta {\delta }^{15}N)}^{2}}$$

For measurements of paramagnetic relaxation enhancements, BEST-TROSY experiments were recorded on MTSL labeled protein in paramagnetic and diamagnetic states. PREs were evaluated by the intensity ratio *I*_para_/*I*_dia_, where *I*_para_ and *I*_dia_ are the resonance intensities of the MTSL labeled protein in its paramagnetic and diamagnetic state, respectively.

GdmCl titration experiments were conducted by recording of ^1^H-^15^N-HSQC spectra on ^15^ N-labeled NCS-1 with concentrations of GdmCl ranging from 0 to 1 M in increments of 0.1 M. The samples were prepared by mixing of various ratios of two samples containing NCS-1 with and without 1 M GdmCl. To accommodate the high ionic strength and resulting long pulse lengths, 3 mm tubes were used in the titration. Amide ^1^H and ^15^N chemical shifts changes > 0.08 and 0.4, respectively, were fit separately to the following equation:

6 $$\delta \left([\mathrm{C}]\right)={\delta }_{0}+\left(\frac{\left[\mathrm{C}\right]}{\left[\mathrm{C}\right]+{K}_{\mathrm{d}}}\right)\left({\delta }_{\mathrm{F}}-{\delta }_{0}\right)+\mathrm{m}\left[\mathrm{C}\right],$$

where $\delta \left(\mathrm{C}\right)$ is the chemical shift change at GdmCl concentration [C], ${\delta }_{0}$ = $\delta (0)$, ${\delta }_{\mathrm{F}}$ = $\delta (\infty )$ and m is a baseline correction factor.

For temperature titrations experiments ^1^H-^15^N-HSQC spectra were recorded on ^15^N-labeled NCS-1 in the range from 25–37 ℃ in increments of 2 ℃.

### Evolutionary sequence analysis and bioinformatics

NCS-1 homologs were identified and aligned using HHBlits [59]. For the purpose of our analysis, we defined homologs as “having a tail” if non-gap residues aligned with positions 183–185. This was based on the importance of positions 183–185 in our experimental truncation and mutagenesis studies.

Conservation was calculated using the consensusMatrix command from the R Biostrings package [60]. Sequence logos were made using WebLogo [61]. Properties of the different tails were calculated using Agadir (helicity) [62], IUpred (disorder) [63], and using the hydropathy score of Kyte and Doolittle [64].

### Surface plasmon resonance

We determined the binding kinetics for the NCS-1•PIK-1 interaction with SPR using a Biacore T200™ system (GE Healthcare). In this setup, we immobilized FL NCS-1 and Δ180 in different flow cells using thiol chemistry, taking advantage of the endogenous single unpaired cysteine (Cys38) present in NCS-1, thus yielding a well-defined and oriented surface tethering. In brief, we activated carboxylates of a CM4 sensor chip (GE Healthcare) using 50 mM N-hydroxysuccinimide (NHS) and 200 mM N-ethyl-*N*-(3-(diethylamino)propyl)-carbodiimide in H_2_O and the resultant activated NHS-ester was subsequently reacted with 80 mM 2-(2-pyridinyldithio)ethaneamine in 0.1 M Na-borate (pH 8.5) creating a surface linked reactive disulfide. This surface was finally reacted with NCS-1 by injecting 0.2 mg/ml NSC-1 in 50 mM Na-acetate, 5 mM CaCl_2_ (pH 5.5) at low flow 5 µl/min for 10 min. High concentrations of NCS-1 were needed to compensate for the lack of electrostatic pre-concentration of NCS-1 (due to its low pI 4.6) and the unfavorable pH for the chemical reaction. Notwithstanding these limitations, we obtained a coupling yield of 292 RU (13 fmols/mm^2^) for NCS-1 FL and 664 RU (32 fmols/mm^2^) for NCS-1 Δ180. Injection of 50 mM L-cysteine in 0.1 M acetate buffer, 1 M NaCl (pH 4.0) deactivated remaining activated disulfides.

The fragment of PIK1 (residues 121–172) was N-acetylated and C-amidated and purchased from KJ Ross (Denmark) purified by reversed phase HPLC to a final purity of 95%. NCS-1•PIK1_121-172_ interactions were measured at 25 °C by multi- or single-cycle protocols on a Biacore T200™ system [65] using a flow of 50 µl/min running buffer (10 mM HEPES, 150 mM NaCl, 5 mM CaCl_2_, 0.05% (v/v) P20, pH 7.4). In the multicycle protocol, serial twofold dilutions of PIK1_121-172_ (range 10 nM–2.6 µM) in running buffer were injected individually for 600 s (association) in running buffer followed by a 1000-s dissociation phase. Two consecutive injections of 10 mM EDTA in 1 M NaCl regenerated the chip after each concentration of PIK1_121-172_. In the single-cycle protocol, five serial twofold dilutions of PIK1_121–172_ were injected without intervening regenerations and the sensor chip was first regenerated after a 1000-s dissociation following the last injection.

Fitting of the double blank referenced data set by non-linear regression to a bimolecular interaction model assuming pseudo-first-order kinetics yielded the association (*k*_on_) and dissociation (*k*_off_) rate constants, the *K*_D_ (*k*_off_/*k*_on_), as well as the binding capacity (*R*_max_) as follows:

7 $$\frac{\mathrm{dR}}{\mathrm{dt}}={k}_{on}\left[{\mathrm{c}}_{\mathrm{a}}\right]\left({\mathrm{R}}_{\mathrm{max}}-\mathrm{R}\right)-{k}_{\mathrm{off}}R,$$

where *c*_a_ is the injected PIK1 concentration, *R*_max_ is the maximum response level in resonance units (RU), and *R* represents the observed signal in RU. In general, 1 RU represents a surface density of 1 pg protein/mm^2^).

We also determined *K*_D_ at steady-state reaction kinetics as follows:

8 $${R}_{\mathrm{eq}}=\frac{{R}_{\mathrm{max}}\left[{c}_{\mathrm{a}}\right]}{{K}_{\mathrm{D}}+\left[{c}_{\mathrm{a}}\right]},$$

where *R*_eq_ is the response (in RU) at equilibrium.

Standard deviations (shown as ±) refer to variation in the parameters derived from five individual measurements at different concentrations for non-equilibrium analysis, whereas it includes all nine concentrations measured at steady state conditions.

The evaluation software supplied with the instrument was used for global fitting (BiacoreT200 Evaluation™ 3.0).

## Electronic supplementary material

Below is the link to the electronic supplementary material.

Supplementary file1 (PDF 2651 kb)
