# Supplementary material for: Disorder in a two-domain neuronal Ca2+-binding protein regulates domain stability and dynamics using ligand mimicry
Source: Cell Mol Life Sci. 2020 Sep 16;78(5):2263–78. doi: 10.1007/s00018-020-03639-z (PMC7966663; doi:10.1007/s00018-020-03639-z)
Supplement: Supplementary file 1 — Supplementary file1 (PDF 2651 kb) [file 18_2020_3639_MOESM1_ESM.pdf]

SUPPLEMENTARY INFORMATION

Disorder in a two-domain neuronal Ca^{2+}-binding protein regulates domain stability and dynamics using ligand mimicry

Lasse Staby[1&], Katherine R. Kemplen[1&], Amelie Stein[1], Michael Ploug[2,3], Jane Clarke[4], Karen Skriver[1], Pétur O. Heidarsson[5], Birthe B. Kragelund[1*]

1. SUPPLEMENTARY FIGURES AND FIGURE LEGENDS

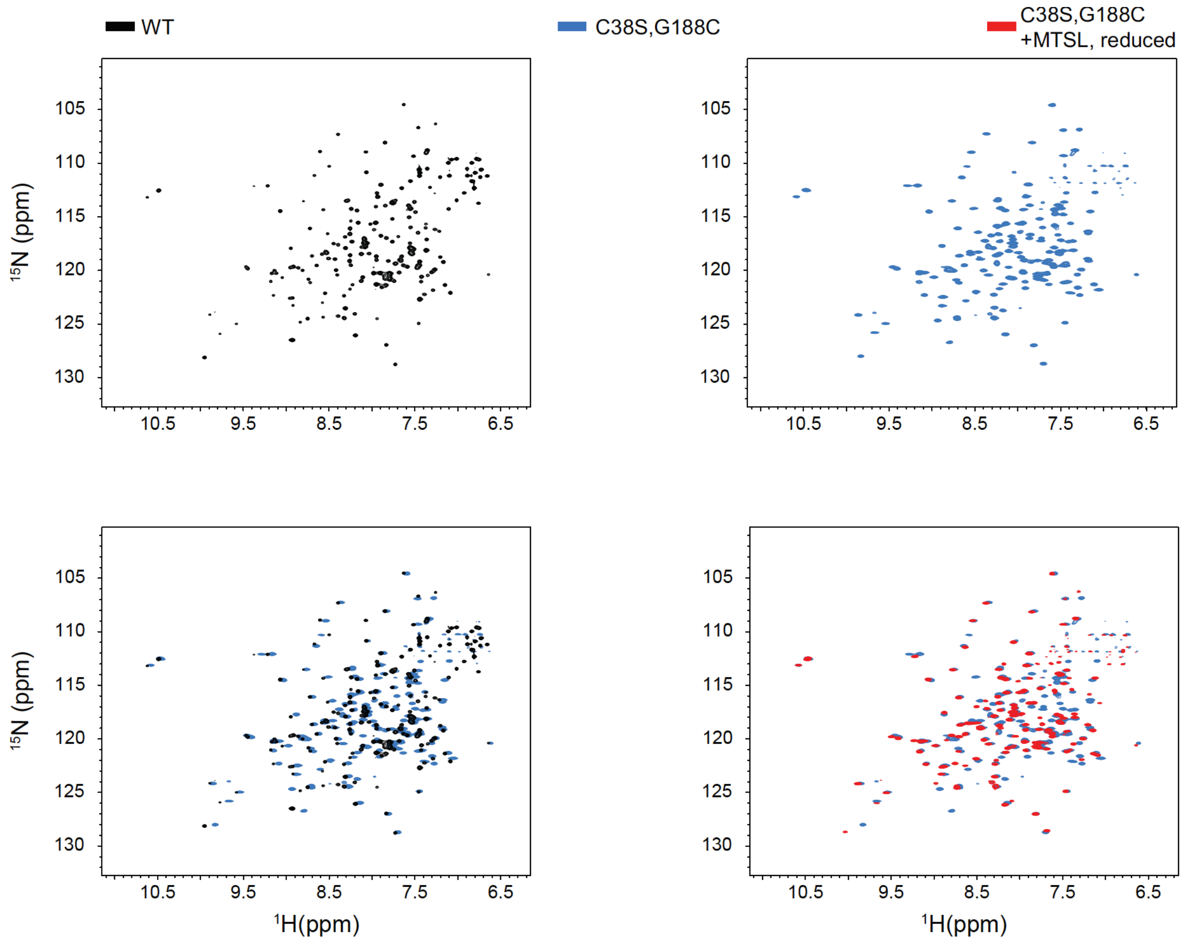

Fig. S1: Supporting data for MTSL-labelled experiments. Comparison of spectra for WT NCS-1 (black), NCS-1 C38S G188C (blue) and reduced NCS-1 C38S G188C +MTSL (red).

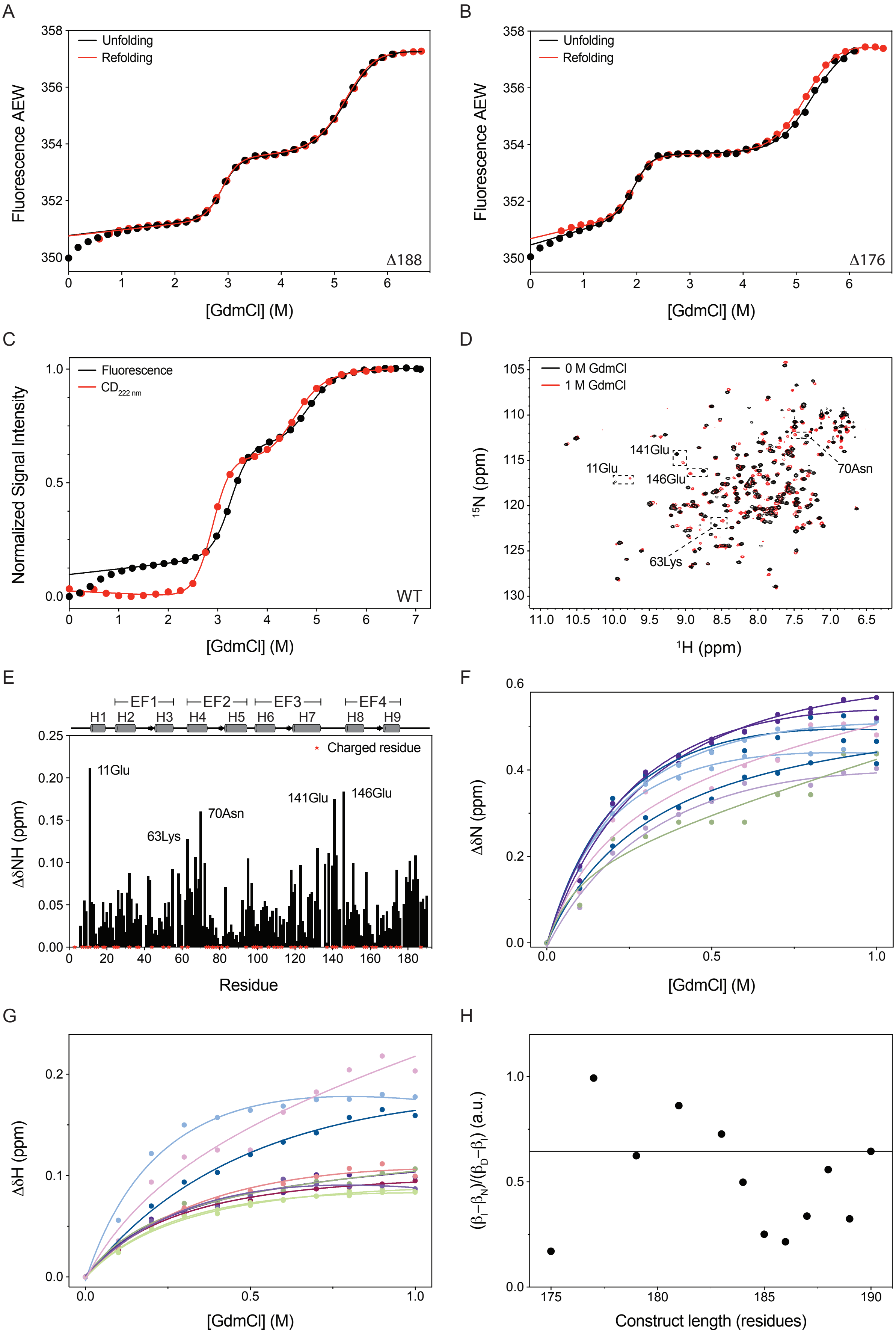

A
Unfolding
Refolding
Fluorescence AEW
[GdmCl] (M)
Δ188
B
Unfolding
Refolding
Fluorescence AEW
[GdmCl] (M)
Δ176
C
Fluorescence
CD 222 nm
Normalized Signal Intensity
[GdmCl] (M)
WT
D
0 M GdmCl
1 M GdmCl
141Glu
11Glu
146Glu
70Asn
63Lys
15N (ppm)
1H (ppm)
E
EF1
EF2
EF3
EF4
H1 H2 H3 H4 H5 H6 H7 H8 H9
Charged residue
11Glu
63Lys
70Asn
141Glu
146Glu
ΔδNH (ppm)
Residue
F
ΔδN (ppm)
[GdmCl] (M)
G
ΔδH (ppm)
[GdmCl] (M)
H
(βI−βN)/(βD−βI) (a.u.)
Construct length (residues)

Fig. S2: Supporting data for stability measurements. **A** and **B** Examples of the global fitting of unfolding (black) and refolding (red) data for two of the variants Δ188 and Δ176. **C** A comparison of unfolding data recorded by fluorescence (black) and CD spectroscopy (red) at 222 nm of WT NCS-1. **D** and **E** $^{1}H,^{15}N$- HSQC spectra of FL NCS-1 in the absence (black) and presence (red) of 1 M GdmCl, and the resulting amide chemical shift perturbations, respectively. The five residues with largest perturbations have been indicated in both figures. **F** and **G** GdmCl binding curves determined from amide and amide proton chemical shift changes, respectively. Only shifts greater than 0.4 ppm or 0.08 ppm for ^{15}N and ^{1}H were considered. **H** Ratios of the amplitude changes for the N- and C-domain unfolding transitions. The black line indicates the ratio for FL NCS-1.

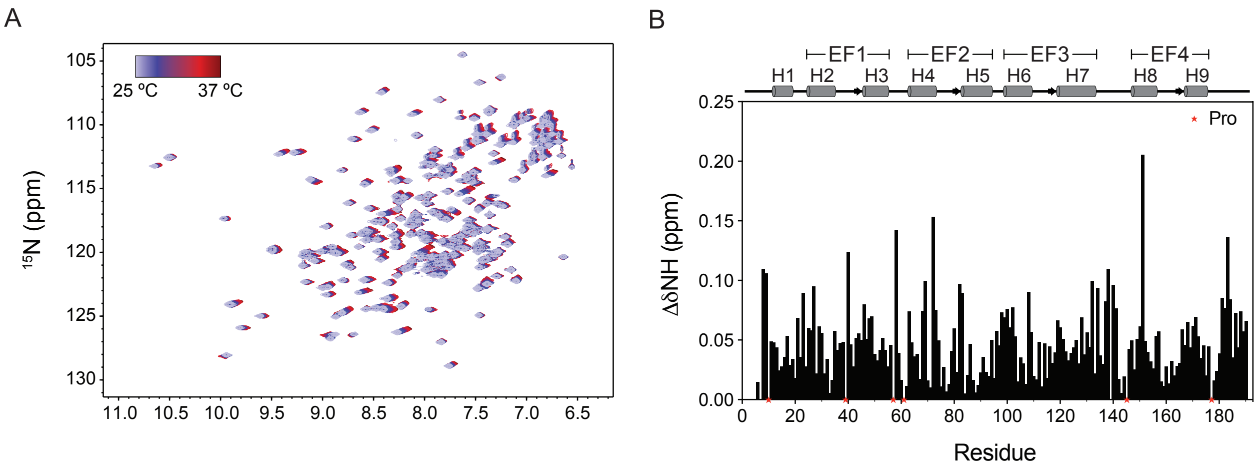

Fig. S3: **A** $^{1}H,^{15}N$- HSQC spectra of FL NCS-1 recorded in the temperature range from 25 ºC to 37ºC in increments of 2 ºC. **B** The resulting combined amide chemical shift perturbations (25 - 37 ºC).

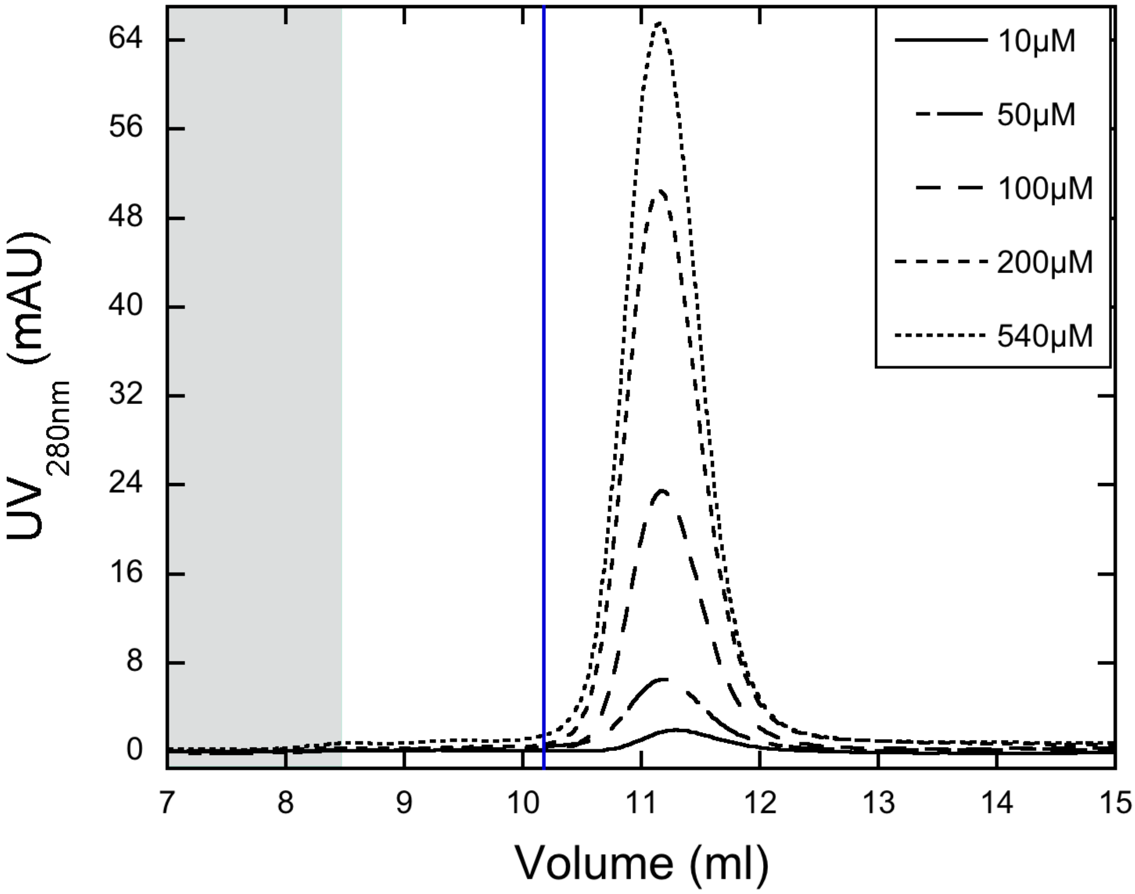

Fig. S4 Analytical size exclusion chromatography showing that the dominant species does not change with increasing concentration. Grey area indicates the void volume, blue line indicates the elution volume of β-lactamase (29kDa).

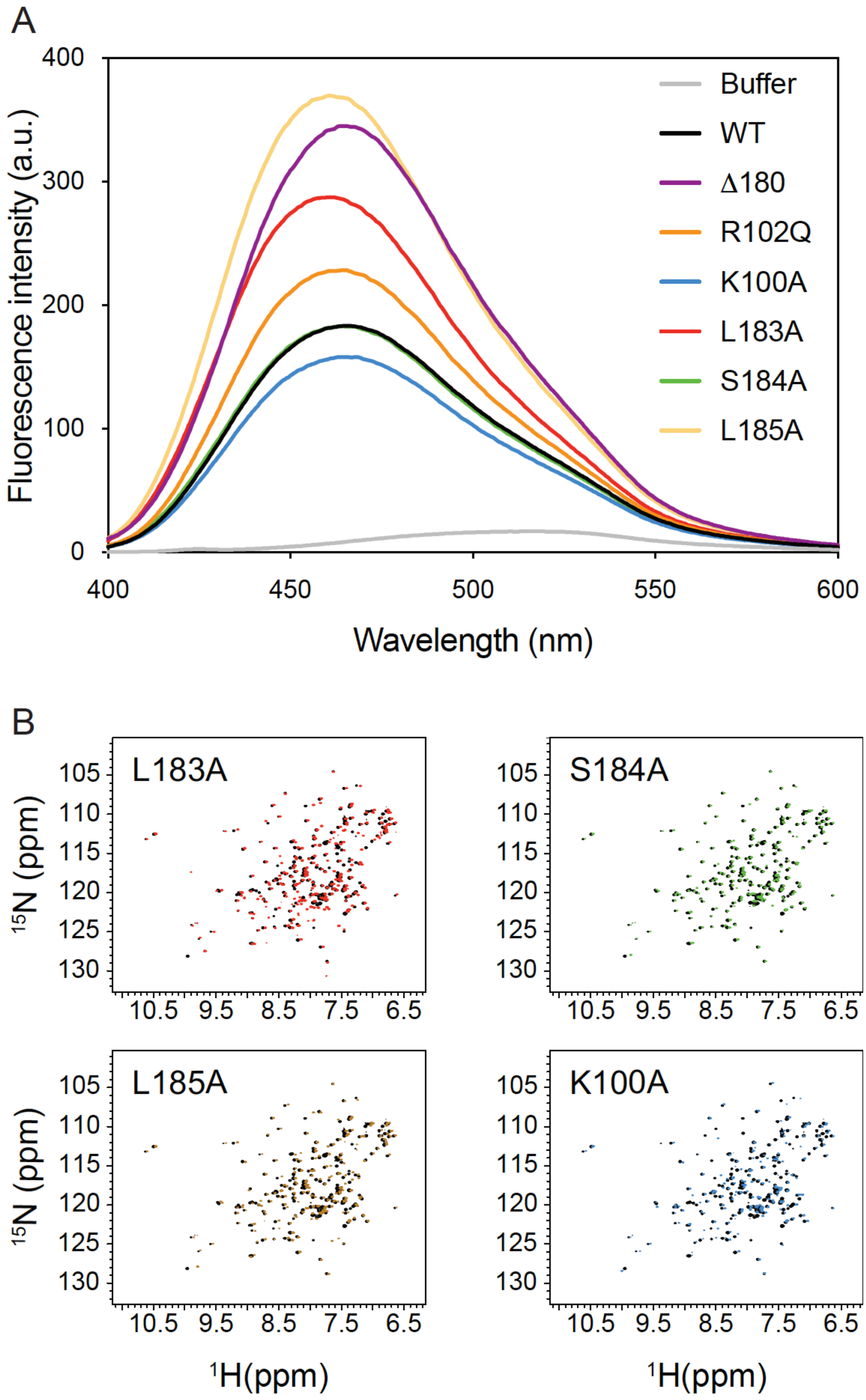

Fig. S5 A ANS-binding assay showing buffer without protein (grey), WT NCS-1 (black) and NCS-1 Δ180 (purple) and single point mutations L183A (red), S184A (green), L185A (yellow) and K100A (blue) and R102Q (orange). **B** Spectral analysis of single point mutation variants of NCS-1 with L183A (red), S184A (green), L185A (orange) and K100A (blue). Wild-type NCS-1 peaks in black.

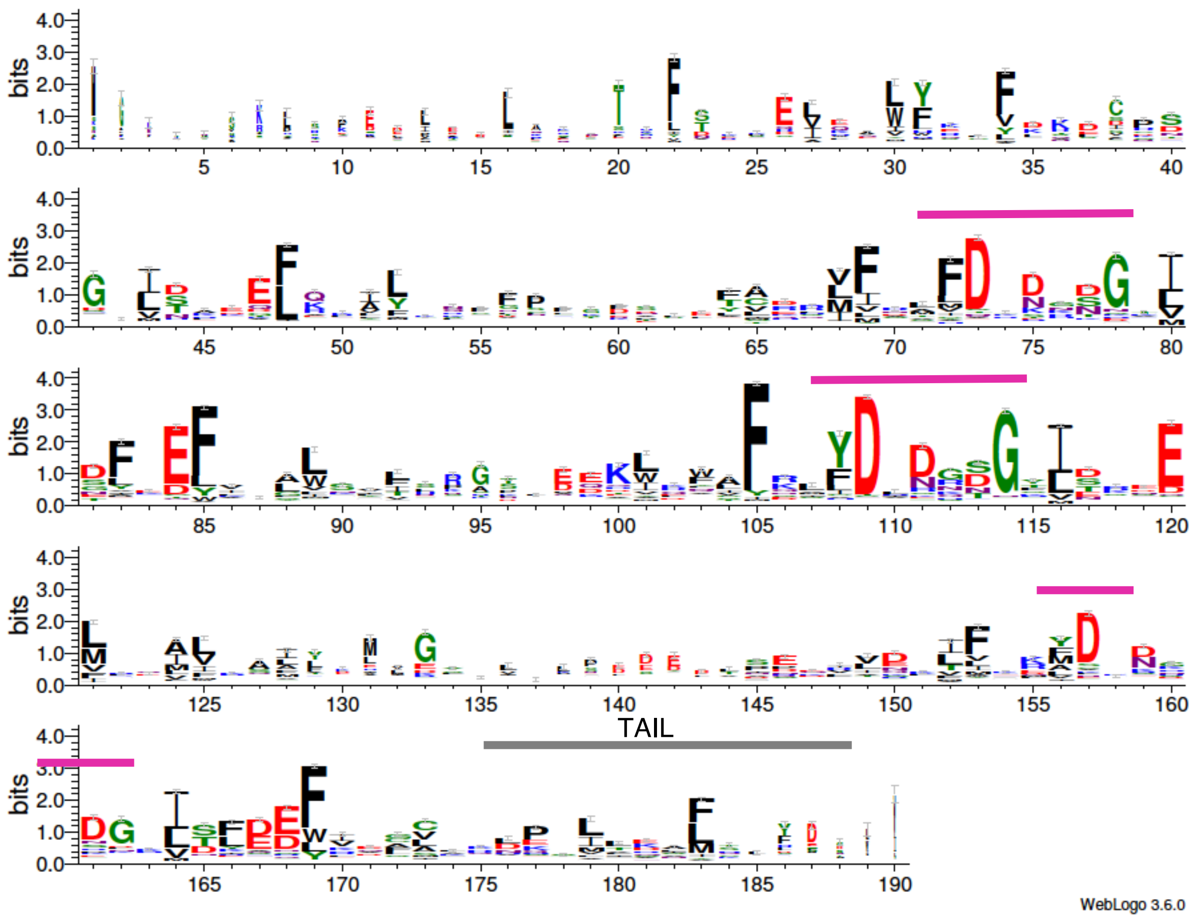

Fig. S6 Sequence logo of the >23.000 tail-carrying Ca^{2+}-sensing family members. The three Ca^{2+} binding sites are identified from the [DxDxxGxΨ]-motifs of the EF-hands (magenta bars), with the two middle sites more pronounced, and the first site not always present. Figure generated using WebLogo3.6.0 (1).

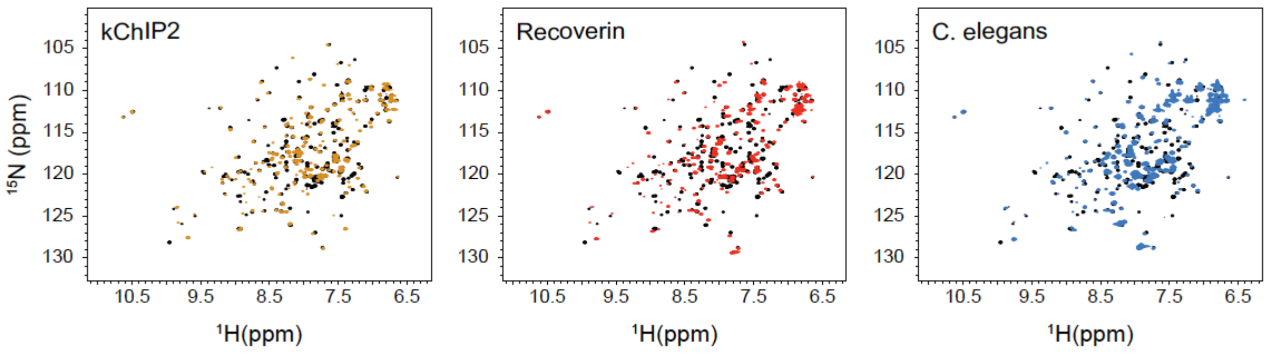

Fig. S7 Spectral analysis of NCS-1 tail chimera by ^{1}H,^{15}N HSQCs. All three spectra of the variants are overlaid the spectrum of WT NCS-1 (black) for comparison.

2. SUPPLEMENTARY TABLES

Table S1. Properties of NCS subfamily members

Protein	Tail Length	PDB code	Known tail structure
hNCS-1	15	2LCP (human, NMR)	disordered
cNCS-1	14	No structure in PDB	-
KChIP2	15	No structure in PDB (homologous structure KChIP-1 1S1E (human, X-ray)	- (homologous structure: helix (2))
recoverin	22	2D8N (human, X-ray); 1J5A (Bos Taurus, NMR)	Last 13 residues disordered (3)

Table S2. Properties of the tails in NCS-1 tail-swap variants

Construct tail	Tail Length	Helicity (%)	GRAVY score	Overall charge	Charged residues (%)	Average IUPred score
hNCS-1	15	0.41	0.71	-2	13	0.42
cNCS-1	14	1.39	-0.77	0	43	0.60
KChIP2	15	2.45	-0.18	-2	27	0.59
recoverin	22	3.13	-1.04	+3	41	0.65

References:

1. Crooks GE, Hon G, Chandonia J-M, Brenner SE (2004) WebLogo: a sequence logo generator. *Genome Res* 14(6):1188–90.
2. Scannevin RH, et al. (2004) Two N-Terminal Domains of Kv4 K+ Channels Regulate Binding to and Modulation by KChIP1. *Neuron* 41(4):587–598.
3. Ames JB, et al. (1997) Molecular mechanics of calcium-myristoyl switches. *Nature* 389(6647):198–202.
